# Optimizing thyroid AUS nodules malignancy prediction: a comprehensive study of logistic regression and machine learning models

**DOI:** 10.3389/fendo.2024.1366687

**Published:** 2024-11-06

**Authors:** Yuan Cao, Yixian Yang, Yunchao Chen, Mengqi Luan, Yan Hu, Lu Zhang, Weiwei Zhan, Wei Zhou

**Affiliations:** ^1^ Department of Ultrasound, Ruijin Hospital, Shanghai Jiao Tong University School of Medicine, Shanghai, China; ^2^ Department of Ultrasound, Zhongshan Hospital (Xiamen Branch), Fudan University, Xiamen, Fujian, China

**Keywords:** thyroid nodules, risk of malignancy, atypia of undetermined significance (AUS), machine learning (ML), logistic regression, prediction model, BRAF V600E

## Abstract

**Background:**

The accurate diagnosis of thyroid nodules with indeterminate cytology, particularly in the atypia of undetermined significance (AUS) category, remains challenging. This study aims to predict the risk of malignancy in AUS nodules by comparing two machine learning (ML) and three conventional logistic regression (LR) models.

**Methods:**

A retrospective study on 356 AUS nodules in 342 individuals from 6728 patients who underwent thyroid surgery in 2021. All the clinical, ultrasonographic, and molecular data were collected and randomly separated into training and validation cohorts at a ratio of 7: 3. ML (random forest and XGBoost) and LR (lasso regression, best subset selection, and backward stepwise regression) models were constructed and evaluated using area under the curve (AUC), calibration, and clinical utility metrics.

**Results:**

Approximately 90% (321/356) of the AUS nodules were malignant, predominantly papillary thyroid carcinoma with 68.6% BRAF V600E mutations. The final LR prediction model based on backward stepwise regression exhibited superior discrimination with AUC values of 0.83 (95% CI: 0.73-0.92) and 0.80 (95% CI: 0.67-0.94) in training and validation, respectively. Well calibration, and clinical utility were also confirmed. The ML models showed moderate performance. A nomogram was developed on the final LR model.

**Conclusions:**

The LR model developed using the backward stepwise regression, outperformed ML models in predicting malignancy in AUS thyroid nodules. The corresponding nomogram based on this model provides a valuable and practical tool for personalized risk assessment, potentially reducing unnecessary surgeries and enhancing clinical decision-making.

## 1 Introduction

The optimal management of thyroid nodules with indeterminate cytology, as classified under the 2023 Bethesda System for Reporting Thyroid Cytopathology (TBSRTC), poses a challenge in clinical practice (1). These indeterminate nodules encompass categories such as atypia of undetermined significance (AUS), follicular neoplasm (FN), and suspicious for malignancy (SUSP), each carrying a variable risk of malignancy from 6% to 94.2%, often influenced by institutional variations or calculation methods (2–6). Despite advancements in high resolution ultrasonography, molecular testing, and other modern diagnostic techniques, no consensus has been reached on the management of AUS nodules. Therefore, efficient risk assessment and intervention strategies are calling for.

Molecular testing has gained prominence in diagnosing indeterminate thyroid nodules and two molecular profiles have been identified: BRAF V600E-like and RAS-like (7, 8). Its applications extend from determining the presence of malignancy to offering prognostic insights and guiding therapeutic decisions (9–11). The BRAF V600E mutation, with its relatively high prevalence and specificity in papillary thyroid carcinoma (PTC), holds particular significance, especially in East Asian populations (12, 13). In China, it has been adopted as a routine element in pre-operative liquid-based biopsy following FNA in the majority of clinical centers. However, the diagnostic efficacy of BRAF V600E mutation alone in AUS nodules is not superior, possibly resulting from variant histopathology, particularly in nodules without the BRAF V600E mutation, where ruling out malignancy is challenging. The combination of demography, imaging, and molecular testing is likely to improve diagnostic accuracy. Therefore, adjunct preoperative assessments are still highly desirable. To the best of our knowledge, studies published to date specifically focusing on predicting the malignancy risk for category III nodules remain limited.

Recently, artificial intelligence (AI) has undergone rapid transformation in the medical field, with deep learning and machine learning emerging as the main algorithms. Numerous studies have been conducted to assess the nature of thyroid nodules and nodule classification using deep learning with ultrasonographic images as input (14, 15). Machine learning is another strategy for diagnosing or predicting thyroid nodules, given its distinctive discrimination efficiency (16). It harnesses data and algorithms to mimic the way that humans learn and make predictions or classifications (17, 18). Moreover, machine learning algorithms have emerged as an alternative to conventional logistic regression analysis for clinical risk prediction models. Despite the extensive exploration of AI in thyroid domain, there is scarce literature addressing the most challenging cohort, the AUS thyroid nodules (16). To date, no reported study has undertaken a comparison of the prediction performance between conventional logistic regression (LR) models and machine learning models (ML) in AUS thyroid nodules.

This study aims to investigate the potential of a multi-faceted approach, incorporating US findings, clinical data, biological features, and genetic information, to predict the risk of malignancy in AUS thyroid nodules. Through a comparative analysis of prediction performance between conventional logistic regression models and machine learning models, our goal is to develop a comprehensive prediction model. The final model will be presented in the form of a nomogram, providing clinicians with a practical tool for assessing AUS thyroid nodules following ultrasound-guided fine needle aspiration (US-FNA).

## 2 Methods

### 2.1 Study design and participants

**Figure 1:**
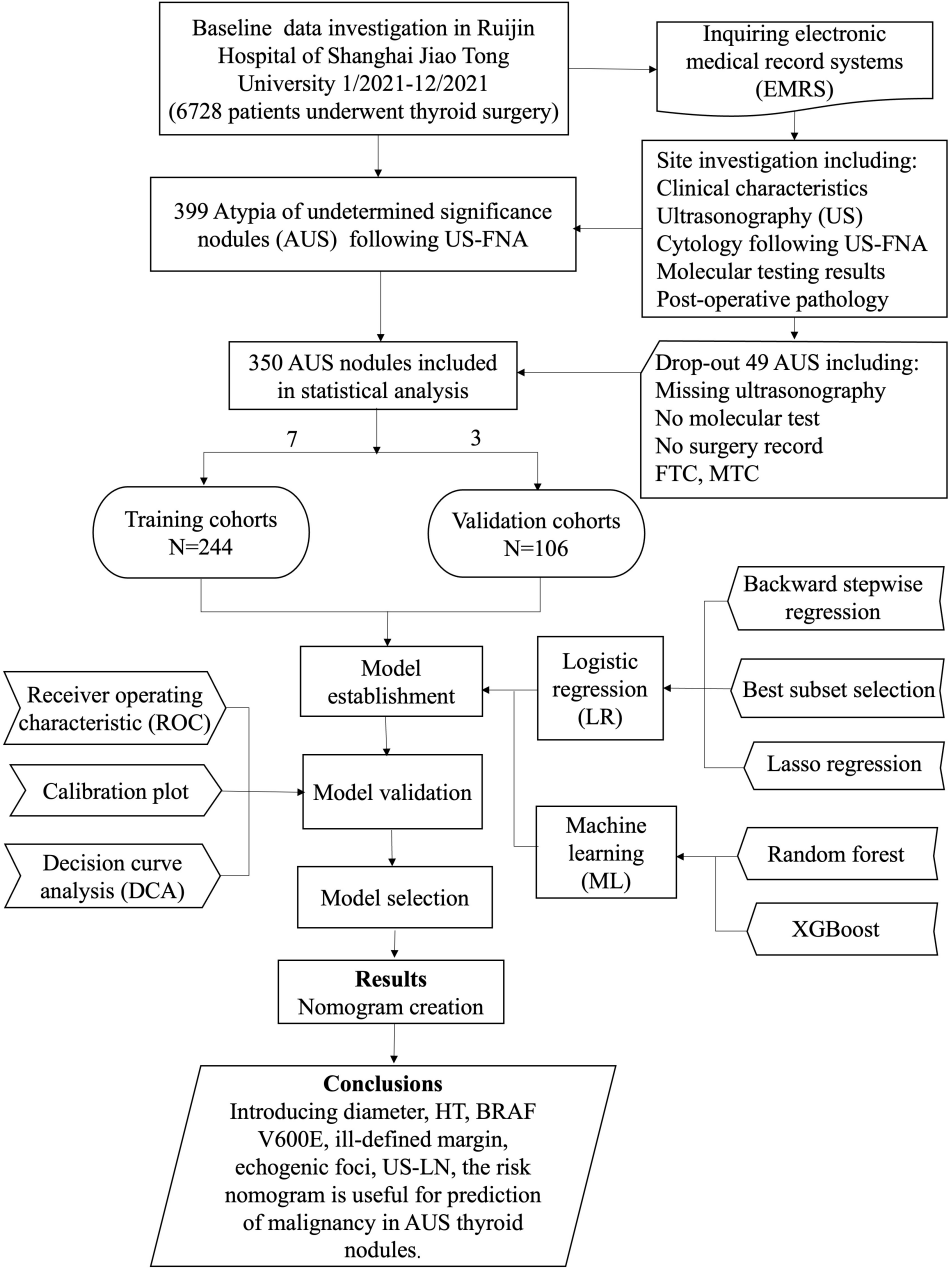
Flow diagram of study design. HT, Hashimoto’s thyroiditis; US-LN, suspected cervical lymph nodes on the US; MTC, medullary thyroid carcinoma; FTC, follicular carcinoma.

This retrospective, single-center study involved consecutive patients who underwent thyroidectomy or thyroid lobectomy at Ruijin Hospital of Shanghai Jiao Tong University between January and December of 2021. The study protocol received approval from the Ethics Committee of Ruijin Hospital. All eligible patients were informed about the use of their data for study and had the option to decline to participate. Informed consent was obtained through a signed agreement before undergoing US-FNA and surgery. Inclusion criteria were: i) age> 18 years; ii) thyroid nodule classified as AUS following FNA cytology; iii) patients who underwent pre-operative US and molecular tests; iv) patients with documented surgery records and final pathology. Exclusion criteria comprised: i) refusal to participate; ii) patients who had undergone thyroid nodule ablation, radioactive iodine, or any other interventional procedure before surgery. The data meeting inclusion criteria were randomly separated into training and validation cohorts at a ratio of 7: 3. Both conventional LR and ML algorithms were applied to create the prediction models in the training dataset, followed by validation in the validation dataset. The final prediction model was chosen based on their performance, complexity, generalization capabilities, and practicality. Various metrics, including discrimination, calibration, and the clinical utility and value of the final prediction, were used for a comprehensive view. Additionally, a nomogram was developed for a graphical computation of the final prediction model. This study adhered to the Transparent Reporting of a multiple prediction model for Individual Prognosis or Diagnosis (TRIPOD) guidelines (19). The workflow of this study is depicted in **Figure 1**.

### 2.2 Ultrasonography

Preoperative neck US was performed in all patients using commercial diagnostic US equipment from various manufacturers (Mindray, Philips, GE Healthcare, Siemens, and HITACHI) with a high-frequency (5-14 MHz) linear array transducer. Settings such as depth, gain, and others were standardized as much as possible across all gray-scale and Color Doppler US examinations. Imaging of the neck, including transverse and longitudinal scanning of the thyroid glands, nodules, and bilateral lymph nodes, was all digitally stored in our hospital’s picture archiving and communication system. Two radiologists reviewed and documented all sonographic characteristics of thyroid nodules using the terminology outlined in the I-TIRADS (20). Additionally, specific details such as size, echotexture, ultrasonography of thyroid parenchyma suggesting Hashimoto’s thyroiditis (US-HT), and suspected cervical lymph nodes on US (US-LN) were also recorded. Any disagreements were resolved by discussion or by consultation with a third radiologist.

### 2.3 Cytological assessment and molecular testing following US-FNA

Nodules classified as C-TIRADS 4a or higher grades, or those exhibiting signs of suspected lymph nodes, were recommended for FNA (21). The targeted nodule underwent re-evaluation to determine its location, nearby vessels, and establish a proper needle path before aspiration. Following the guidelines (22), a 23 G needle was inserted through the skin into the nodule, guided by real-time US. Once the needle tip was positioned within the nodule, suction was applied to the needle, which rapidly moved back and forth within the nodule to obtain multiple samples of cells. The collected specimen was smeared and preserved in the liquid-based cytology preservative solution (Hangzhou HealthSky Biotechnology Co., Ltd).

Cytologists examined and analyzed the cells and cellular structures referring to the second edition of the Bethesda System for Reporting Thyroid Cytopathology (23). Molecular testing was done to provide more precise information about the nature of these thyroid nodules. Several molecular tests were available in our institution, including BRAF V600E, Ras, and TERT.

### 2.4 Prediction model establishment and evaluation

For the conventional LR model, several variable selection methods were applied to the training dataset for the best predictor features, including least absolute shrinkage and selection operator regression (LASSO), best subset selection, and backward stepwise regression, with all potential variables as input. Additionally, two popular machine learning algorithms, namely Random Forest (RF) and Extreme Gradient Boosting (XGBoost), were constructed to create a predictive model. To assess the discrimination capabilities of LR and ML models, the receiver operating characteristic area under the curve (ROC AUC) was used on both datasets. The forest plot was introduced to display the estimated coefficients (odds ratios) and their confidence intervals for various predictor variables in the LR model. The reliability and accuracy of the model were evaluated through a calibration curve, which compares the model’s predicted outcomes or probabilities to the actual observed outcomes. This curve provides valuable insights into the model’s calibration performance. The decision curve analysis (DCA) curve was utilized to evaluate the clinical utility and value of the model, offering a more comprehensive assessment of model performance than traditional metrics like sensitivity and specificity (24). DCA helped identify the most appropriate threshold probability for the model, considering user preferences for minimizing false positives or false negatives in the decision-making process and optimizing model application in practical scenarios. Finally, a nomogram was developed based on the final predictive model. This graphical tool simplifies the estimation of individual risk or probabilities based on the model’s predictor variables (25).

### 2.5 Statistical analysis

All analyses were conducted in R, version 4.3.0 (R Project for Statistical Computing). The population characteristics were described using frequencies and proportions for categorical variables, and medians and interquartile ranges (IQR) for continuous variables. The chi-square test and Fisher’s exact test were employed to compare categorical variables between the training and validation datasets. Sixteen features concerning patient demographics (age, gender), ultrasonography, and molecular testing (BRAF V600E) were recorded as potential variables to predict pathology outcomes. The “glmnet”, “leaps”, and “MASS” packages in R was used to select predictors by LASSO, best subset selection, and backward stepwise regression in the training dataset, respectively. Select the optimal model with the minimum criterion value (minimum AIC, BIC, or maximum adjusted R-squared). The “ROSE” package in R was applied to aid the task of binary classification in the presence of rare classes in ML establishment with over-sampling based on a bootstrap technique. The “caret” package in R was introduced to define optimal hyperparameters in the RF model using 5-fold cross-validation. The XGBoost model was fine-tuned by incorporating regularization parameters like gamma, lambda (L2 regularization term), and alpha (L1 regularization term) into a grid search to identify the optimal hyperparameters. For all tests, P<0.05 was considered statistically significant.

## 3 Results

### 3.1 Analysis of baseline information

From January to December 2021, a total of 6728 patients underwent thyroid surgery, and 399 thyroid nodules were diagnosed as AUS following US-FNA. Among them, 342 individuals (279 female and 77 male) with 356 AUS nodules and complete records were enrolled in this study. Approximately 90% (321/356) of the nodules were malignant, with the majority being PTC (315/321), along with a few cases of medullary thyroid carcinoma (5/321) and follicular carcinoma (1/321). Within the PTC cases, 68.6% (216/315) had BRAF V600E mutations. The remaining 10% (35/356) were benign lesions. The median age was 43.5 years (IQR 35.0-53.0), and the median nodule size was 5.1mm (IQR 3.98-7.93). Five medullary and one follicular carcinoma were excluded during predictors selection. As described in **Table 1**, these baseline features showed significant similarity in the training (n=244) and validation (n=106) cohorts (p>0.05).

**Table 1:** Baseline characteristics of patients with AUS thyroid nodules in training and validation cohorts.

Variables	Total *N*=350	Training *N*=244 (70%)	Validation *N*=106 (n=30%)	P value
Age (median [IQR])	44.0 [35.0, 53.0]	44.0 [36.0, 54.0]	43.5 [34.25, 52.0]	0.683
Diameter (median [IQR])	5.1 [3.9, 7.6]	5.1 [4.1, 7.5]	5.3 [3.9, 8.47]	0.679
Gender (%)				
Female	277 (79.1)	191 (78.3)	86 (81.1)	0.645
Male	73 (20.9)	53 (21.7)	20 (18.9)	
Direction of growth (%)				
Wider-than-tall	94 (26.9)	68 (27.9)	26 (24.5)	0.605
Taller-than-wide	256 (73.1)	176 (72.1)	80 (75.5)	
Irregular margin (%)				
No	33 (9.4)	22 (9.0)	11 (10.4)	0.840
Yes	317 (90.6)	222 (91.0)	95 (89.6)	
Ill-defined margin (%)				
No	244 (69.7)	171 (70.1)	73 (68.9)	0.920
Yes	106 (30.3)	73 (29.9)	33 (31.1)	
Composition (%)				
Solid	329 (94.0)	229 (93.9)	100 (94.3)	1.00
Predominately solid	21 (6.0)	15 (6.1)	6 (5.7)	
Echogenicity (%)				
Markedly hypoechoic	34 (9.7)	25 (10.2)	9 (8.5)	0.799
Mildly hypoechoic	307 (87.7)	212 (86.9)	95 (89.6)	
Isoechoic	9 (2.6)	7 (2.9)	2 (1.9)	
Echotexture (%)				
Homogeneous	247 (70.6)	175 (71.7)	72 (67.9)	0.556
Heterogeneous	103 (29.4)	69 (28.3)	34 (32.1)	
Echogenic foci (%)				
No echogenic foci	198 (56.6)	142 (58.2)	56 (52.8)	0.793
Microcalcifications	104 (29.7)	70 (28.7)	34 (32.1)	
Macrocalcifications	22 (6.3)	15 (6.1)	7 (6.6)	
Micro-macro foci	26 (7.4)	17 (7.0)	9 (8.5)	
Color Doppler Flow Imaging (%)				
None	3 (0.9)	2 (0.8)	1 (0.9)	0.773
Slight	312 (89.1)	215 (88.1)	97 (91.5)	
Moderate	16 (4.6)	13 (5.3)	3 (2.8)	
Abundant	19 (5.4)	14 (5.7)	5 (4.7)	
Capsule contact (%)				
No	132 (37.7)	92 (37.7)	40 (37.7)	1.00
Yes	218 (62.3)	152 (62.3)	66 (62.3)	
US-HT (%)				
No	255 (72.9)	182 (74.6)	73 (68.9)	0.329
Yes	95 (27.1)	62 (25.4)	33 (31.1)	
US-LN (%)				
No	278 (79.4)	195 (79.9)	83 (78.3)	0.842
Yes	72 (20.6)	49 (20.1)	23 (21.7)	
Pathology				
Benign	35 (10.0)	24 (9.8)	11 (10.4)	1.00
Malignant	315 (90)	220 (90.2)	95 (89.6)	
Hashimoto thyroiditis (%)				
No	287 (82.0)	202 (82.8)	85 (80.2)	0.667
Yes	63 (18.0)	42 (17.2)	21 (19.8)	
BRAF V600E mutation (%)				
No	125 (35.7)	85 (34.8)	40 (37.7)	0.690
Yes	225 (64.3)	159 (65.2)	66 (62.3)	

AUS, atypia of undetermined significance; US-HT, ultrasonographic of thyroid parenchyma suggesting Hashimoto’s thyroiditis; US-LN, suspected cervical lymph nodes on US.

### 3.2 Machine learning model

**Figure 2:**
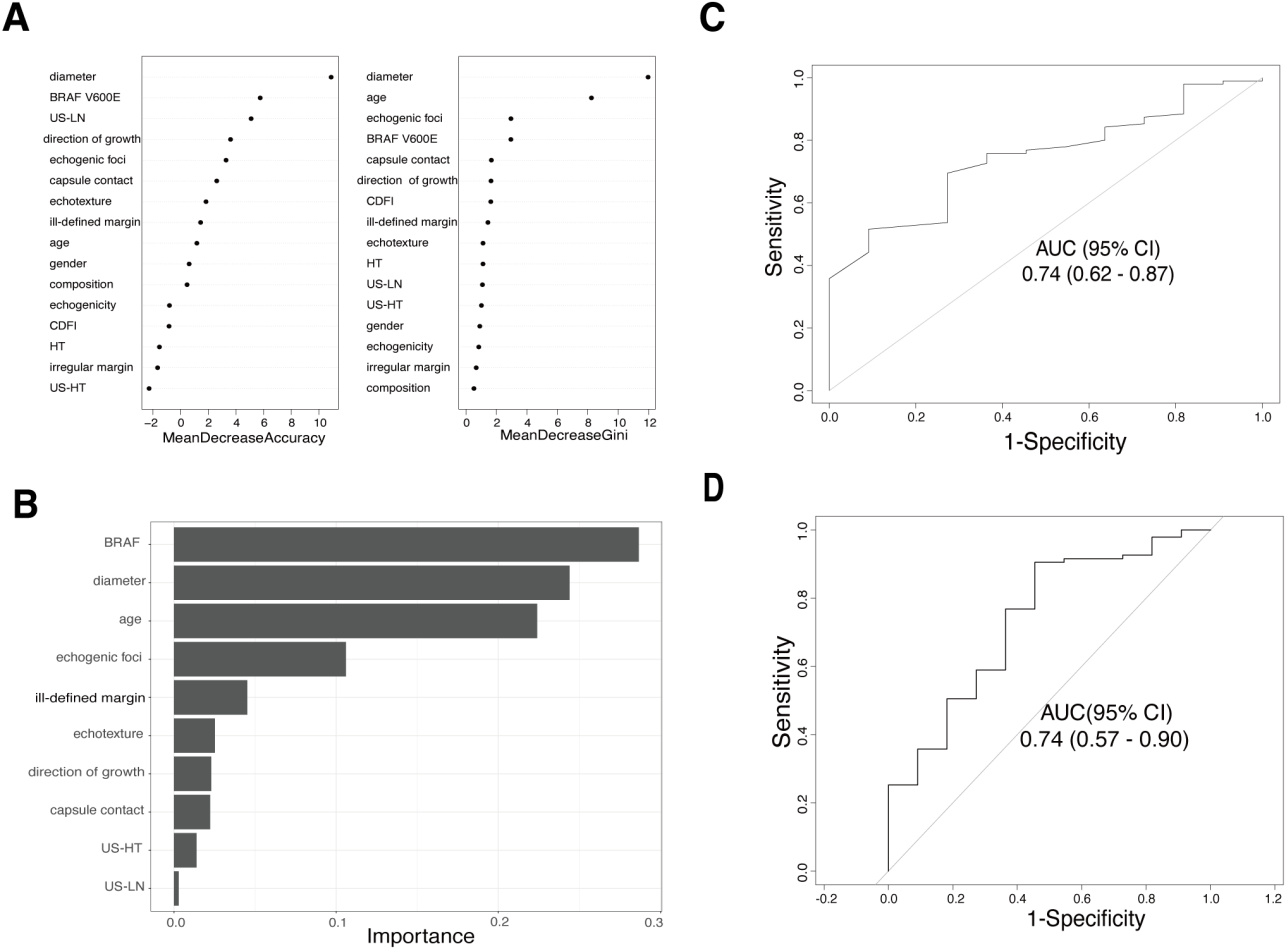
The relative contribution of predictor variables and the area under the receiver operating characteristic curve (AUC) in two Machine learning models. **(A)** Variable importance ranking plot for random forest model. **(B)** Out-of-bag variable importance ranking for the XGBoost model. **(C)** ROC curve for the random forest model in the validation cohort. **(D)** ROC curve for the XGBoost model in the validation cohort.

**Figures 2A, B** illustrate the relative importance of potential features in the RF and XGBoost models based on the training cohort. The performance of the random forest and XGBoost models were evaluated using AUC values on the validation set, resulting in values of 0.74 (95% CI: 0.62-0.87) for random forest (**Figure 2C**) and 0.74 (95% CI: 0.57-0.90) for XGBoost (**Figure 2D**). The RF model was figured with 100 trees (ntree = 150) and considered two random features at each split (mtry = 4). This configuration was fine-tuned based on testing and adjustments, ultimately resulting in the lowest estimation error rate of out-of-bag samples (OBB=9.02%). The XGBoost model was constructed with specific hyperparameters in terms of minimizing log loss.

### 3.3 Logistic regression model

**Figure 3:**
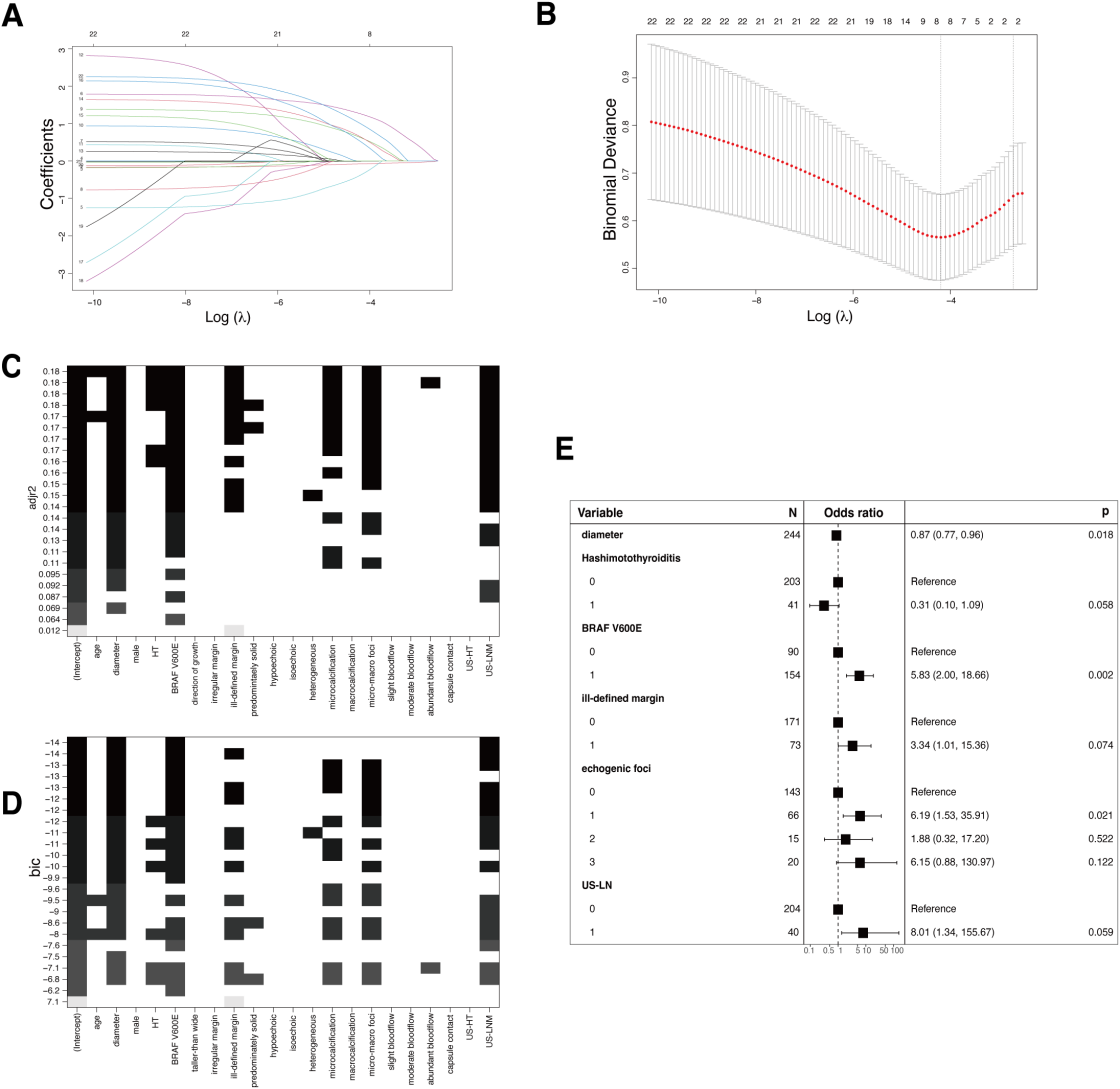
Conventional logistic regression (LR) models. **(A)** Variable screening via the LASSO binary logistic regression model is depicted, showing the coefficient profiles of 22 variables in the training cohort. **(B)** The 10-fold cross-validation process is visualized for the selection of the tuning parameter (λ) in the LASSO model. Vertical dotted lines denote the points of minimum mean square error (λ=0.015) and the standard error of the minimum distance (λ=0.067). **(C)** The process of variable selection with best subset selection regression on Adjusted R-Squared (adjr2). Seven variables were selected when the adjr2 was maximized, achieving the balance between explanatory power and model simplicity. **(D)** Best subset selection on the Bayesian Information Criteria (BIC). The same seven variables as selected based on adjr2 were chosen, as the BIC suggests a more efficient model in terms of explanatory power and complexity. **(E)** The forest plot shows the independent predictors in backward stepwise regression. Each square represents the point estimate of the effect size (e.g., odds ratio), the horizontal line indicates the 95% confidence interval, and the vertical dotted line represents the line of no effect, where the odds ratio equals 1.

**Figure 4:**
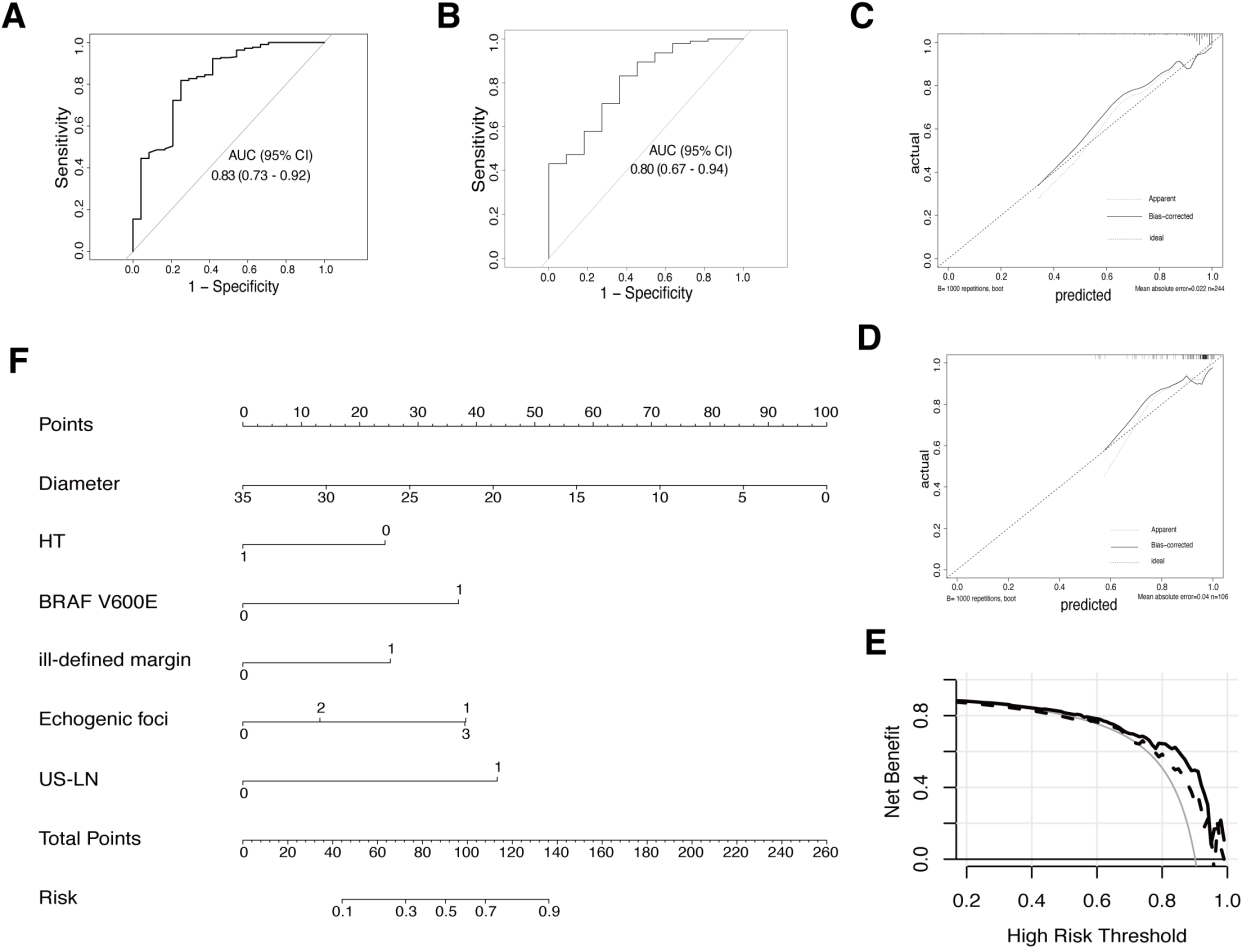
The final model (LR based on backward stepwise regression) evaluation and display. **(A)** ROC curve for final prediction model in the training cohort. **(B)** ROC curve in the validation cohort. **(C)** Calibration plot for this model in the training cohort. **(D)** The calibration plot in the validation cohort. **(E)** Decision curve analysis (DCA) for the model in both training (solid line) and validation cohorts (dashed line). **(F)** A nomogram was developed based on the final prediction model to estimate the risk of malignancy for AUS thyroid nodules. HT (0: without Hashimoto’s thyroiditis, 1: the presence of Hashimoto’s thyroiditis; BRAF V600E (0: wild type, 1: mutation); ill-defined margin (0: clear margin, 1: ill-defined margin); Echogenic foci (0: no echogenic foci; 1: micro-calcification; 2: macro-calcification; 3: both micro & macro calcification); US-LN (0: nonsuspicious lymph-node, 1: suspicious lymph node).

In the training set, independent predictors of the LR prediction model were selected from 16 potential variables through lasso regression (**Figures 3A, B**), best subset selection (**Figures 3C, D**), and backward stepwise regression. The final predictable variables were diameter, Hashimoto’s thyroiditis, BARF V600E, ill-defined margin, echogenic foci, and US-LN as described in the forest plot (**Figure 3E**). The AUC values of the model based on backward stepwise regression in the training cohort were 0.83 (95% CI: 0.73-0.92) (**Figure 4A**), and 0.80 (95% CI: 0.67-0.94) in the validation cohort (**Figure 4B**), respectively. As illustrated in **Figures 4C, D**, this model demonstrated superior calibration performance in both cohorts.

### 3.4 Model selection

Both the RF and XGBoost models showed moderate AUC values on the validation set, indicating their ability to discriminate between malignancy and benignity, but without exceptionally high discrimination performance compared with LR models. Considering aspects such as model performance, complexity, generalization capabilities, and practicality, the LR based on backward stepwise regression prediction model was chosen finally. The forest plot depicted that certain variables with P-values exceeding 0.05, such as Hashimoto’s thyroiditis, ill-defined margin, and US-LN, were retained in this logistic regression, as a result of their clinical significance in daily medical practice, and to allow for the interpretation of their sizes.

### 3.5 Comprehensive assessment of the final model

In both the training and internal validation set, the AUROC curve analysis yielded sensitivity values of 0.82 and 0.83, as well as specificity values of 0.75 and 0.64, respectively. The mean absolute error (MAE) for both data sets was 0.022 and 0.04 after performing bootstrapping 1000 times in the calibration plot, indicating that the model’s predicted probabilities closely align with the actual outcomes across different samples (**Figures 4C, D**). The DCA curve was applied to both data sets to evaluate the clinical utility and value of the model. As **Figure 4E** revealed, the utilization of this prediction model within the threshold probability range of 20-95% in the training cohort demonstrated a higher net benefit compared to both the screen-none and screen-all strategies. In the validation cohort, a threshold probability within 55-95% showed a favorable outcome.

### 3.6 Nomogram development

To depict this mathematical model vividly, a nomogram was created by assigning a weighted score to each of the predictors, as shown in **Figure 4F**. The total scores could be calculated as a summary of each predictor’s scores, identified by drawing lines to the points axis. Refer to the risk line, the probability of malignant in AUS thyroid nodules was estimated. (For example, if a patient had Hashimoto’s thyroiditis, a 20mm thyroid nodule with characteristics such as microcalcification and ill-defined margin, and the cervical lymph nodes were normal and AUS without BRAF V600E mutation after US-FNA, they would score 110 points, indicating a 72% estimated malignancy risk).

## 4 Discussion

Thyroid nodules, particularly those categorized as Bethesda III following US-FNA, remain a clinical challenge. Despite the TBSRTC 2023 reclassification of AUS/FLUS as AUS only, along with revised management recommendations, the accurate differentiation between benign and malignant nodules is still crucial for clinical decision-making (1). Therefore, we constructed a model to predict malignancy in AUS nodules, aiming to provide a reference for patients and physicians. Although several studies have been conducted to address this challenge, many have been limited by small sample sizes and a lack of comprehensive postoperative pathology results and other essential features (26–30). In our study, we take a more extensive approach by analyzing a large dataset of AUS nodules, incorporating comprehensive clinical, ultrasound, genetic, and pathological data. The nodules were randomly divided into training and validation cohorts to ensure the robustness of our analysis.

To develop more reliable predictive models for AUS nodules assessment, we considered several key variables selected through various methods, including LR with LASSO, backward stepwise regression, and best subset selection, as well as ML like RF and XGBoost, following a rigorous comparison. Despite the increasing popularity of ML-based models in predictive modeling, our study revealed that the LR-based nomogram exhibited superior performance in predicting malignancy of AUS thyroid nodules in terms of AUC, calibration, and clinical utility. This finding is in line with the claim that ML may not consistently outperform LR for prediction modeling (31, 32). We attempted to rectify the imbalanced training dataset in ML through oversampling and parameter adjustments, but it yielded few improvements. Furthermore, Silke Janitza and Roman Hornung suggest that OOB error may overestimate the true prediction error and raise uncertainty about its use for tuning random forest parameters, which could affect model performance (33). It remains difficult to predict whether LR would consistently outperform random forest on all future data, as we used OOB error for parameter selection.

Notably, in this study, we integrated a broad spectrum of features beyond typical ultrasound parameters outlined in I-TIRADS, recognizing the multifactorial nature of AUS nodules assessment, where clinical, imaging and genetic factors interplay intricately. The independent variables in our final predicting model encompassed diameter, Hashimoto’s thyroiditis, BRAF V600E mutation status, ill-defined margin, echogenic foci, and the suspicion of cervical lymph node metastasis based on US.

With an odds ratio of 0.87 for the nodule diameter, larger AUS nodules following US-FNA seem to have a lower likelihood of malignancy compared to smaller ones. Yoon et al. introduced a nomogram incorporating clinical and ultrasound features to predict malignancy among AUS/FLUS nodules (26). Our study expands beyond typical ultrasound and clinical features, suggesting that the presence of Hashimoto’s thyroiditis (odds ratio = 0.31) might contribute to the malignancy prediction model. In our predictive model, AUS nodules in the context of HT were assigned lower points compared to those without HT. Ultrasonography may be challenging for PTC in the context of HT due to its increased or decreased parenchyma echogenicity and coarsened echotexture with nodular margins. The association of HT and PTC has been a topic of controversy. On one hand, some studies have indicated that HT may elevate the risk of thyroid malignancy (34, 35). Several mechanisms have been proposed that TSH stimulation, proto-oncogenes such as BRAF mutations, and RET/PTC rearrangements may promote cancer development or growth. Chronic lymphocytic thyroiditis in HT may induce inflammation factors fueling cancer cell proliferation, while neoplastic cells could trigger a chronic inflammatory response as well (36). On the other hand, conflicting findings exist, with some reports confirming that the presence of Hashimoto’s thyroiditis does not affect the risk of malignancy in thyroid nodules of category III (37). These differing findings reveal the complexity of thyroid nodule assessment coexisting with HT.

Simultaneously, the BRAF V600E mutation plays a vital role in assisting malignancy prediction. As the most extensively studied mutation in thyroid cancer, it has been reported to possess an approximate specificity of 100% in PTC (diagnostic role), leading to a significant reduction in unnecessary thyroid surgeries (7, 38). However, the isolated presence of BRAF V600E mutation is unlikely to discover the full picture of thyroid carcinogenesis due to its poor sensitivity. A meta-analysis revealed that its value as a single screening test alone in AUS is limited, with approximately 40% sensitivity in indeterminate nodules (39). Possible reasons for this limitation include the reported histopathology of carcinomas in AUS, which encompasses classic and tall cell variants of PTC, follicular thyroid carcinoma, Hurthle cell carcinoma, squamous cell, lymphoma, and others. Genetic mutations involved in these nodules also include BRAF K601E, RET-PTC, PAX8/PPARγ, RAS, etc. Incorporating a wider range of molecular markers could improve the predictive power of the models and provide deeper insights into the underlying biological mechanisms. Słowińska-Klencka et al. emphasized the importance of a holistic approach in managing category III nodules, highlighting the potential of combining miRNAs, BRAF V600E mutation, and EU-TIRADS (European Thyroid Imaging and Reporting Data System) to support clinical decision-making (40). Zhao et al. have also reported a 10.1% false-positive and a 7.1% false-negative rate of BRAF V600E mutations in thyroid FNA specimens, with a great improvement in diagnostic performance when combined with FNA cytology (41).

The nomogram, serving as a clear and intuitive visual tool, allows clinicians to easily understand and estimate probabilities without performing complex calculations. It was constructed based on the prediction model for assessing thyroid nodules. In cases where thyroid nodules are confirmed as indeterminate by US-FNA, this nomogram can be utilized as a reference for prediction.

Our models were developed with a wide range of data, including US findings, clinical data, biological features, and genetic information, and involved a thorough comparative analysis of prediction performance between conventional logistic regression models and machine learning models for a comprehensive prediction model. However, the interobserver variability and the evolving nature of medical guidelines remain inherent challenges, especially in ultrasonographic features collected. Nevertheless, many artificial intelligence (AI) technologies have emerged and are increasingly being applied to medical imaging (42, 43). Various deep learning-based AI models have demonstrated strong performance in feature extraction, serving as valuable aids for thyroid management (44). In our study, we specifically focused on patients with AUS nodules after FNA, who were initially assessed by ultrasonography and subsequently managed through surgery rather than active surveillance. All the ultrasonographic features were reported baesd on the established guidelines and clinical experience. Although LR based on backward stepwise regression was chosen finally, it is of great importance to continue research and refinement of the prediction models as new knowledge and guidelines emerge.

While this prediction model was established to reduce the need for unnecessary surgeries and alleviate patient anxiety associated with indeterminate nodules, several limitations should be considered. Firstly, although we analyzed a larger dataset of AUS nodules compared to many previous studies, a larger sample size could further enhance the generalizability of our findings. Secondly, due to a single-center, retrospective study, our results may be subject to selection bias. The availability of data, as well as potential missing or incomplete information could have have influenced our results. Variations in clinical practices among institutions and regions may also play a role. The high malignancy rate in our cohort might have influenced the developments of the prediction model. Prospective multi-center investigations are needed to establish stronger causal relationships and external validity. Additionally, our focus on the BRAF V600E mutation, limited exploration of other prevalent molecular factors in thyroid cancer, and the observed discrepancies regarding HT and thyroid cancer risk highlight the need for more extensive molecular and mechanism research in this field. Future studies should aim to incorporate these additional mutations to improve the comprehensive and accuracy of the prediction models. Lastly, the potential for interobserver variability in ultrasound feature interpretation and the evolving nature of medical guidelines emphasize the need for ongoing research in this dynamic area.

## 5 Conclusion

In conclusion, our predictive model, incorporating US features, clinical data, and genetic information, offers a practical tool for assessing AUS thyroid nodules. Moreover, the model demonstrated promising performance and outperformed the machine learning algorithm. While this research represents a significant advancement in personalized medicine, reducing the ambiguity associated with indeterminate nodules and ultimately improving patient care, it highlights the need for larger-scale studies and further molecular investigations in this domain.

## Data Availability

The original contributions presented in the study are included in the article/supplementary material. Further inquiries can be directed to the corresponding authors.
